# Addressing hypersensitivity in density functional theory for reliable machine learning

**DOI:** 10.1038/s41524-026-02191-x

**Published:** 2026-06-19

**Authors:** L. Arnstein, J. Wetherell, R. Lawrence, P. J. Hasnip, M. J. P. Hodgson

**Affiliations:** 1 School of Physics, Engineering and Technology, University of York, York, UK; 2 The European Theoretical Spectroscopy Facility, Louvain-la-Neuve, Belgium; 3 Physics Unit, Faculty of Engineering and Natural Sciences, Tampere University, Tampere, Finland

**Keywords:** Materials science, Mathematics and computing, Physics

## Abstract

In principle, machine learning (ML) can be used within density functional theory to obtain any electronic property of a time-independent many-body system from its ground-state electron density alone. However, when the target quantity (the output) is highly sensitive to small variations in the density (the input), accurately learning the mapping between them is highly challenging in practice. We identify this ‘hypersensitivity’ in two classes of density functionals that contain observables central to most electronic structure calculations: (1) any observable that varies subject to a global constant shift in the external potential (such as the total energy); (2) any observable of the *N*-electron system that depends on the properties of the (*N* + 1)-electron system (such as the electron affinity). We demonstrate that the accuracy with which widely used ML models approximate density functionals in these two classes is significantly limited by the pathological nature of the mapping from the density to the observable, even when the reference level of the external potential is fixed to prevent constant shifts. In addition, we find that providing the external potential as input to the ML model resolves this issue, in some cases reducing the error by up to three orders of magnitude when predicting quantities in these two classes.

## Introduction

Materials science relies on computational models to predict the properties of matter. This involves electronic structure calculations of many-electron systems, for which exact solutions to the Schrödinger equation are generally intractable.

Due to its balance of accuracy and computational efficiency, density functional theory (DFT) has become the most popular method for electronic structure calculations^1^. Formally, DFT is an exact approach to quantum mechanics in which the ground-state electron density, *n*(***r***), replaces the many-body wavefunction as the quantity from which observables are calculated. This formalism is rigorously justified by the Hohenberg-Kohn (HK) theorems^2^, the first of which establishes that the ground-state electron density uniquely determines the external potential, *v*_ext_(***r***), up to an arbitrary additive constant. By extension, *n*(***r***) determines the entire many-body Hamiltonian and, implicitly, all properties derivable from it^3^. While the first HK theorem is restricted to densities associated with an external potential (‘*v*-representable’), the constrained search formalism^4^ extended DFT to all densities that can be derived from an *N*-electron antisymmetric wavefunction (‘*N*-representable’). This ensures that all observables are well-defined functionals of the density^5,6^, even in the case of degeneracy or when the density is not *v*-representable.

The second HK theorem states that there exists a total energy functional *E*[*n*] = *E*_ext_[*n*] + *F*[*n*] which is minimised when *n*(***r***) is the ground-state density. *E*_ext_[*n*] = ∫*v*_ext_(***r***)*n*(***r***)d^3^*r* is the external potential energy. *F*[*n*], which contains the electron-electron repulsion and the kinetic energy, is a universal functional of the density, meaning that it is the same for all systems, independent of *v*_ext_(***r***) ^2,4^. As such, if the exact form of *F*[*n*] was known, the ground-state total energy of any many-body system could be calculated via the variational principle^4^. In practice, expressing *F*[*n*] as a pure density functional (particularly the kinetic energy component^7^) proved to be extremely challenging. This led the original, ‘orbital-free’ vision of DFT to be abandoned in favour of the Kohn-Sham (KS) approach^8^, which relies on single-particle orbitals in addition to the electron density. The orbitals describe an auxiliary system of non-interacting electrons that are constrained to have the same electron density as the real, many-body system. Their kinetic energy, *T*_s_, is computed from the orbitals, side-stepping the problem of finding a density functional of the kinetic energy. As *T*_s_ tends to be close to the true, many-body kinetic energy and closed-form expressions for the Hartree energy and the external potential energy are known, this leaves only a (usually) relatively small unknown component of the total energy—termed the exchange-correlation energy, *E*_xc_[*n*]—to be approximated. Although many existing approximations perform well for specific system classes, the search for more reliable approximations to *E*_xc_[*n*] is ongoing^9^. Moreover, the introduction of orbitals came with a significant computational overhead, limiting KS-DFT to modelling systems of hundreds or at most a few thousand electrons^10^. In contrast, orbital-free DFT could, in theory, simulate millions of electrons.

Although the total energy is the central functional in DFT, *any* property of a time-independent many-body system can, in principle, be expressed as a pure density functional^3,4^. However, owing to a lack of reliable explicit density functional approximations (DFAs) for many physical quantities, the electronic properties of the KS system are employed, despite known limitations. For instance, the KS band (fundamental) gap is often used as an approximation to the real, many-body band gap, even when they differ significantly^11,12^. If accurate DFAs were available for properties such as the band gap, this could significantly accelerate progress in materials science^13^. Unfortunately, traditional methods for constructing DFAs rely on prior knowledge of the functional, which is lacking for almost all properties^14^. In addition, as the DFA for the band gap is likely to be highly non-linear and non-analytic, it may not be feasible to approximate it well with a simple analytic form.

Recently, machine learning (ML) has emerged as a promising approach for developing DFAs^15–17^. ML models learn the relationship between the electron density, *n*(***r***), and an observable, *Q*, via a collection of examples known as training data. A successfully trained model should then be able to generalise to unseen data, accurately predicting the values of *Q*[*n*] corresponding to densities that were not used for training. These predictions can be made significantly faster than the original method used to generate the training data^18,19^, even when non-local descriptors are used. Moreover, ML models such as kernel ridge regression (KRR) and neural networks (NNs) are universal approximators^17^; given sufficient parameters, they can, in theory, approximate any mapping to arbitrary precision. Crucially, these models do not require any prior knowledge of the functional^14^, enabling them to capture complex dependencies between *n*(***r***) and *Q* that may evade human intuition^20^. As such, ML offers an alternative route to attain DFAs for a variety of properties where traditional, analytical tools have fallen short.

A primary goal of ML-DFT is orbital-free DFT^17^, where the hope is that ML can eliminate the need for orbitals and thereby overcome the primary computational bottleneck of contemporary DFT calculations. At a minimum, this requires machine-learning the non-interacting (Kohn-Sham) kinetic energy as a density functional, *T*_s_[*n*] ^20–24^. Alternatively, by explicitly modelling the universal functional *F*[*n*] ^23^, or equivalently the total energy *E*[*n*] ^19,25–29^, the auxiliary KS system can be bypassed altogether. Due to its flexibility, ML also opens up the possibility of modelling a variety of density functionals outside of the familiar energy functionals^14^, such as the electron affinity^13^ and the band gap^13,14,28–31^.

A natural alternative to the electron density is to use the external potential as the ML input to predict these observables^19,26,32,33^. This approach is based on potential functional theory (PFT)^34–36^, a rigorous dual formalism to DFT^17^. Although not all potential → observable mappings are unique (e.g. in the case of ground-state degeneracy, the potential → density mapping is non-injective), many observables are well-defined functionals of the external potential. In these cases, the density and potential should represent equally valid ML inputs, as both contain *all* the information about a *v*-representable system with a given number of particles^2–4^. However, they do not necessarily lead to equally accurate predictions in practice. Ref ^26^ found *n*(***r***) to be the better input for predicting the total energy of molecules. On the other hand, ref. ^19^ found the opposite result for some system classes, with *v*_ext_(***r***) yielding more accurate predictions of the total energy. The results of ref. ^19^ also indicate that the relative effectiveness of using the potential or density depends on the observable being predicted. However, a systematic understanding of the origins of these differences and of the strengths and failure modes of each input is currently lacking. This understanding would facilitate the development of accurate and robust ML models for electronic structure calculations.

In this paper, we identify a fundamental pitfall in using the electron density as the sole ML input to predict observables belonging to two classes (described below), such as the total energy, the electron affinity and the fundamental gap. We show that small variations in the density can correspond to dramatic changes in the value of these quantities. This ‘hypersensitivity’ is inherently challenging from an ML perspective^17,37,38^, requiring complex models and large, high-quality datasets to accurately learn the steep density → observable mapping. We also show that this pitfall is avoided by using the external potential as input to the ML model. The hypersensitivity stems from significant changes in the potential that strongly affect the observable but leave the density largely unchanged; as such, the potential → observable mapping is not hypersensitive and hence more amenable to ML approximation. Our findings can therefore help to inform input selection for a variety of important observables. They also explain several aspects of the results of ref. ^19^, as well as their apparent contradiction with ref. ^26^.

The first class of density functionals in which we identify hypersensitivity corresponds to physical quantities that change value when a non-zero constant, *c*, is added to the external potential. Formally, these quantities are not invariant under a global U(1) gauge transformation of the external potential, hereafter referred to as ‘constant shift dependence’ for simplicity:

### Definition 1

Let the physical quantity *Q* correspond to the potential *v*_ext_(***r***), and, likewise, $\bar{Q}$ correspond to ${\bar{v}}_{{\rm{ext}}}({\boldsymbol{r}})$, where ${\bar{v}}_{{\rm{ext}}}({\boldsymbol{r}})={v}_{{\rm{ext}}}({\boldsymbol{r}})+c$. *Q* is *constant shift dependent* if $\bar{Q}\ne Q$, and *constant shift invariant* if $\bar{Q}=Q$.

**Fig. 1:**
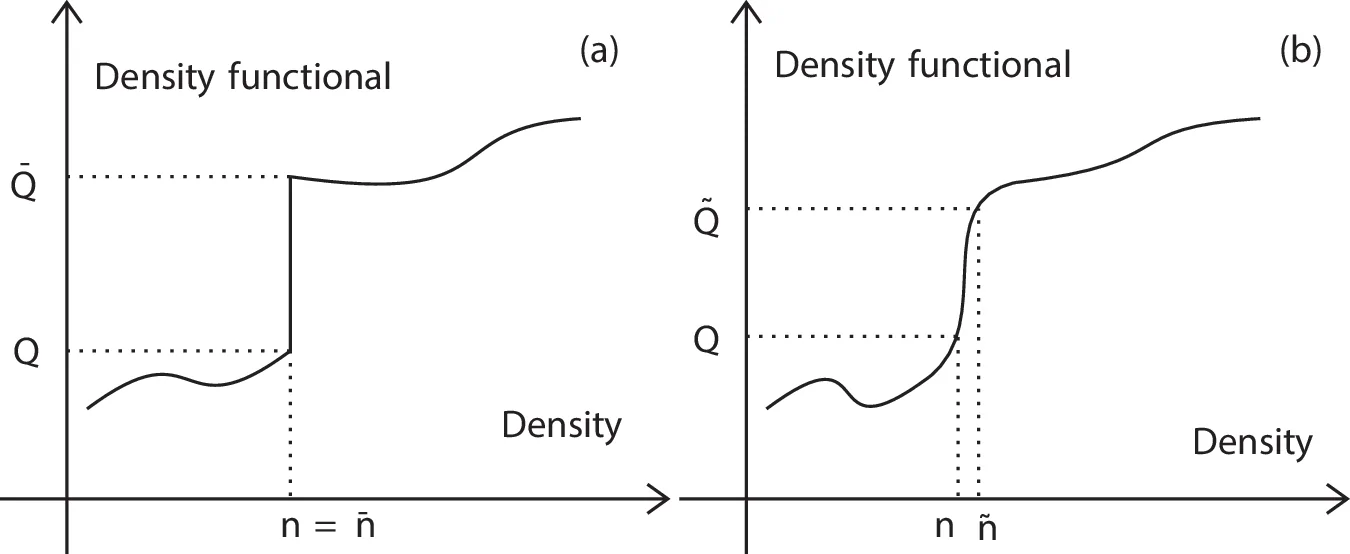
Schematic of a constant-shift dependent density functional. **a** Under a constant shift in the external potential, the quantity jumps in value, but the electron density does not change. As the same input maps to two different outputs, the learning problem is intractable. This situation is avoided by fixing the reference level of the external potential, which prevents constant shifts from occurring. **b** Under a near-constant shift in the external potential, the functional is well-defined but changes so rapidly that it is challenging to machine-learn in this region of density space.

Examples of constant-shift dependent quantities include the total energy, $E,\,(\bar{E}=E+Nc)$, the first ionisation energy, $I,\,(\bar{I}=I-c)$ and the electron affinity, $A,\,(\bar{A}=A-c)$. The electron density itself, on the other hand, is constant shift invariant, i.e. $\bar{n}({\boldsymbol{r}})=n({\boldsymbol{r}})$. Therefore, a constant (spatially uniform) shift in the external potential will cause the same density to map to two different values of a constant-shift dependent quantity, *Q*, as depicted in Fig. 1a. However, this can be resolved by using a fixed reference level for the external potential, such as *v*_ext_(*r* → *∞*) = 0, which prevents any two potentials from differing exactly by a constant over all space. Under this constraint, each density maps to a single value of *Q*.

**Fig. 2:**
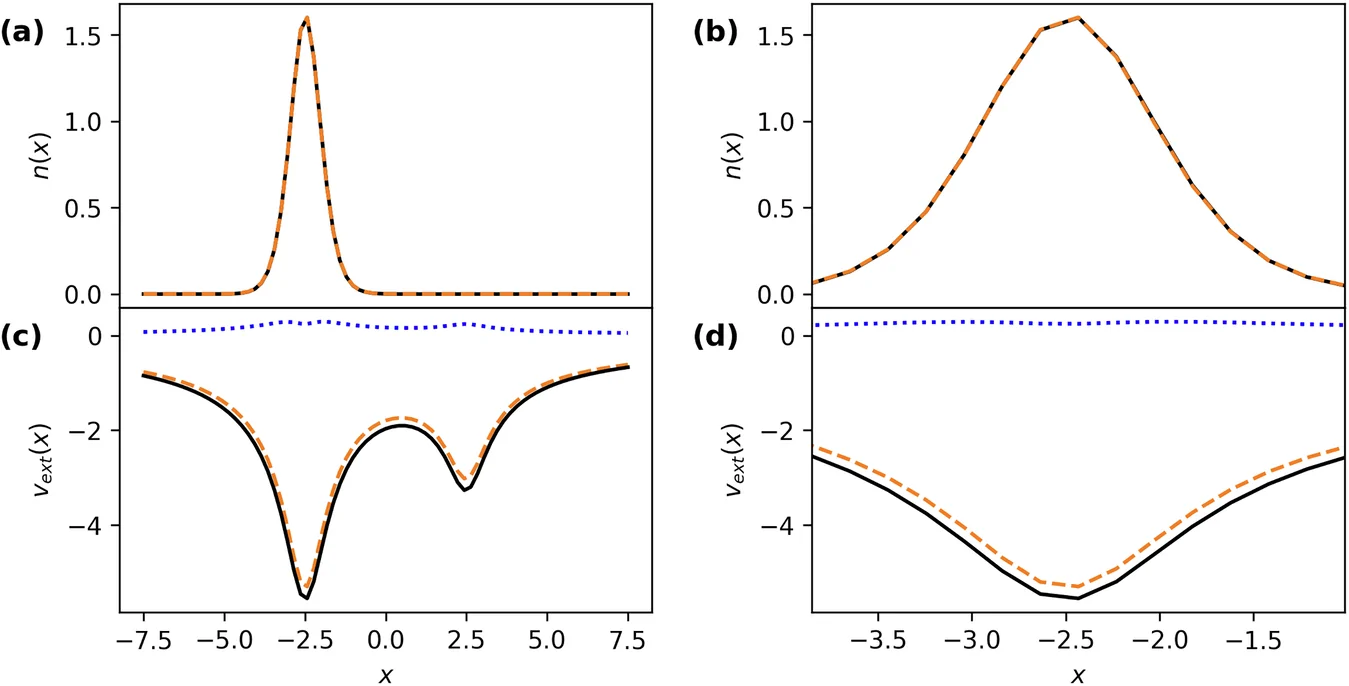
An example pair of systems from our first dataset that illustrates a near-constant shift. In the left panels, they are plotted over the full domain of the 1D grid. The black solid lines and dashed orange lines each correspond to a system (see text for details), for which the external potentials and electron densities are plotted in (**c**) and (**a**), respectively. The blue dotted line shows the difference between the external potentials, *Δv*_ext_(*x*). In (**c**), it can be seen that *Δv*_ext_(*x*) varies over space. The right panels show a close-up to the region where most of the electron density is concentrated; in this region, *Δv*_ext_(*x*) is approximately constant (**d**). Consequently, the electron densities of the two systems are almost indistinguishable (**b**), but they differ substantially in their constant-shift dependent properties—for example, the system represented by solid black lines has a total energy of −7.72 Ha, whereas the other system has a total energy of −7.18 Ha.

However, we note that even when the reference level is fixed, two potentials may differ approximately by a constant in the region where the density is concentrated (for example, see Fig. 2 in section ‘Dataset 1’). In this region, their difference can be described as *Δv*_ext_(***r***) = *c* + *δv*_ext_(***r***), where the spatially varying component, *δv*_ext_(***r***), is small. Note that *δv*_ext_(***r***) could even be 0 over a strict subset of space while still satisfying the fixed reference level. Since *n*(***r***) is constant shift invariant, it only responds to the small, non-constant component of *Δv*_ext_— therefore, the difference between the electron densities of the two systems, *Δn*(***r***), is likely to be very small. On the other hand, the difference in a constant-shift dependent property, *ΔQ*, may be significant, e.g. *ΔE* ≈ *Nc*. *Q*[*n*] is therefore hypersensitive, as similar inputs map to highly disparate outputs (this is illustrated in Figure 1b). In other words, ∣*ΔQ*/*Δn*∣ is very large, because ∣*ΔQ*∣ ≫ ∣*Δn*∣. Conversely, *Q*[*v*_ext_] is not hypersensitive, because *Δv*_ext_(***r***) is not small.

In summary, the reference level of the external potential must be fixed to prevent global constant shifts and ensure a well-defined mapping from the electron density to a constant-shift dependent quantity. However, near-constant shifts, which can occur even with a fixed reference level, cause these quantities to vary rapidly with respect to the electron density, making learning highly challenging. Note that this problem does not affect all quantities that depend explicitly on the external potential, because some such quantities are not constant shift-dependent. For example, the fundamental gap, *E*_*g*_ = *I* − *A*, depends explicitly on the external potential but is constant shift invariant: ${\bar{E}}_{g}=\bar{I}-\bar{A}=I-c-(A-c)=I-A={E}_{g}$. We emphasise that the relevant factor is constant shift dependence, not dependence on the external potential.

**Fig. 3:**
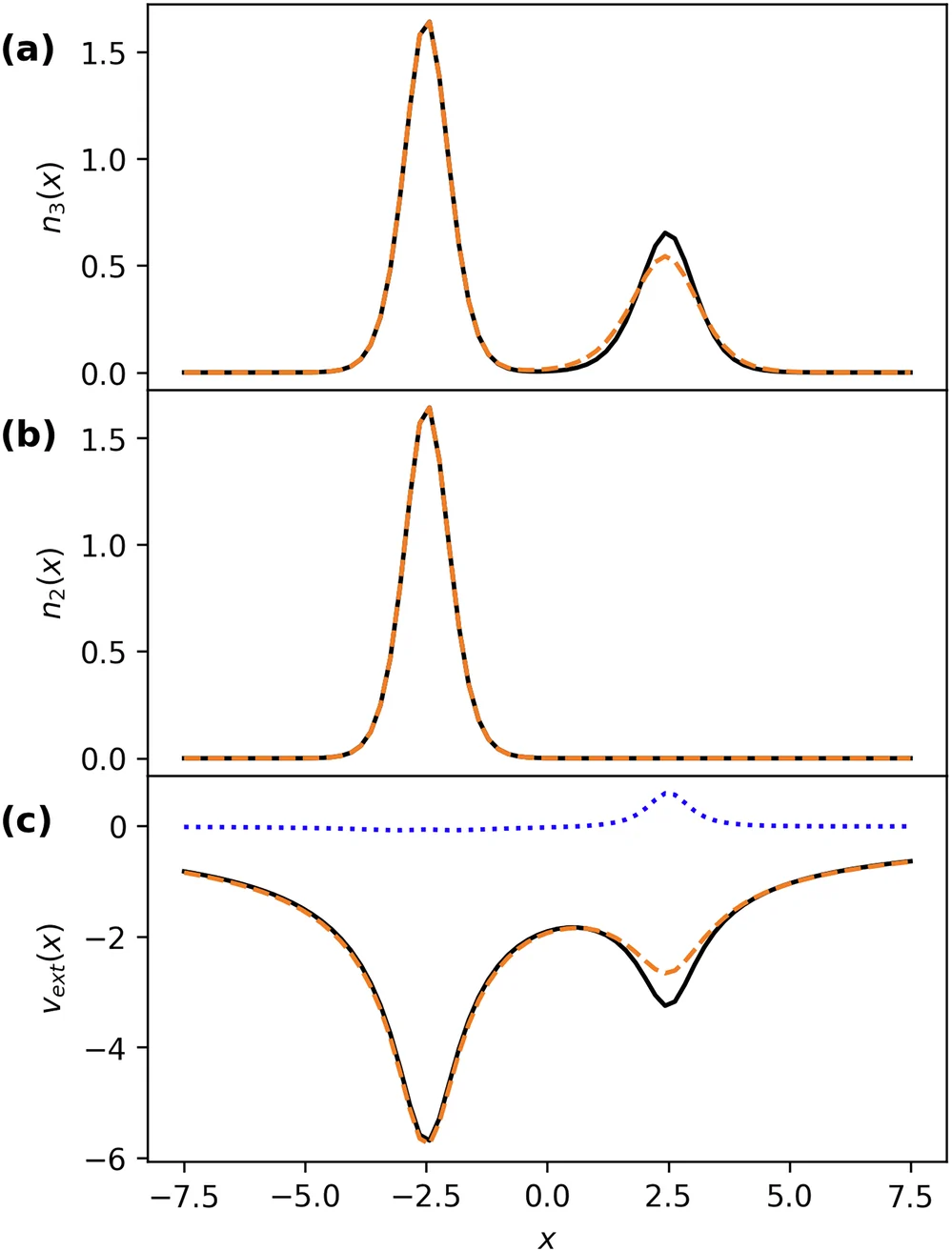
Two systems (one indicated by solid black lines, the other by dashed orange lines—details in the main text) that illustrate how dependence on the properties of the $\left(N+1\right)$-electron system can lead to hypersensitivity in our second class of density functionals. They are both two-electron molecular systems wherein both electrons are confined to the left well (**b**). In this region, the difference between the external potentials of the two systems (**c**) is close to 0, leading to similar electron densities. However, the corresponding three-electron densities (**a**) are spread between both wells, and the potentials differ significantly in the right well. This leads to large differences between the three-electron energies and quantities that depend on them, such as the affinity and fundamental gap. As such, the changes in these properties of the two-electron systems are disproportionate to the small change in the two-electron density.

The second class of hypersensitive functionals that we highlight is functionals of the *N*-electron density that depend on the properties of the corresponding (*N* + 1)-electron system. Examples include the electron affinity, *A*, and the fundamental gap, *E*_*g*_, which both explicitly depend on the total energy of the (*N* + 1)-electron system (*A* = *E*_*N*_ − *E*_*N*+1_ and *E*_*g*_ = *E*_*N*−1_ − 2*E*_*N*_ + *E*_*N*+1_). Since the density of the *N*-electron ground state uniquely determines the external potential, it by extension determines the Hamiltonian and its complete spectra—including the energies of the *N* − 1 and *N* + 1 states. Therefore, the fundamental gap and electron affinity are mathematically well-defined functionals of the *N*-electron ground-state density. However, hypersensitivity arises in these functionals if some change to the external potential significantly affects the (*N* + 1)-electron system, and hence the functional output, but not the *N*-electron system, and hence not the functional input. For example, consider two external potentials that only differ significantly in one region of space; see Fig. 3c in section ‘Dataset 1’. If the *N*-electron densities of both systems are low in this region, they are likely to be insensitive to the difference and hence similar overall. However, if the corresponding (*N* + 1)-electron systems have significant density in the region where the external potentials differ appreciably, properties such as the affinity and fundamental gap will differ significantly for the two external potentials. These quantities are therefore hypersensitive when expressed as functionals of the *N*-electron density alone. In contrast, since the difference between the external potentials of the two systems is substantial, these same properties are *not* hypersensitive when expressed as potential functionals.

Below, we use one-dimensional (1D) model systems to test our hypotheses. We use the iDEA code^39^ to model various systems of two fully interacting electrons subject to double-well molecular potentials by solving the many-body Schrödinger equation numerically. For each system, we calculate the exact many-body electron density *n*(*x*), the total energy *E*, the first ionisation energy *I*, the electron affinity *A*, the fundamental gap *E*_*g*_, and the HK universal functional *F*. We provide examples of hypersensitivity, wherein systems with near-identical electron densities differ substantially in their values of *E* (Class 1), *I* (Class 1), *A* (Classes 1 and 2) or *E*_*g*_ (Class 2). This is measured numerically as finite difference quotients ∣*ΔQ*/*Δn*∣ (see Eq. (3)) on the order of 10^2^. We contrast this with the universal functional, which falls in neither class, and subsequently, ∣*ΔF*/*Δn*∣ is consistently two orders of magnitude smaller. Similarly, we show that (within the dataset) ∣*ΔQ*/*Δv*_ext_∣ does not exceed the order of 1 for all five observables, confirming our hypothesis that the hypersensitivity is avoided by expressing these quantities as potential functionals. We note that while PFT avoids the ‘Class 1’ and ‘Class 2’ hypersensitivity we find in density functionals, potential functionals may still exhibit hypersensitivity in other contexts.

We then train KRR models to predict each physical quantity, first providing the entire (discretised) electron density as the input, and observe several orders of magnitude greater error for all hypersensitive quantities (compared to *F*). When we instead use the external potential as the input, this discrepancy in learning accuracy is not present, with similar accuracy for all observables. These results clearly demonstrate that the hypersensitivity in the density functionals we identified can be a major source of error that is avoided by using the external potential as input. This is further supported by ref. ^19^, who report results that we identify as having greater error for constant-shift dependent density functionals (such as *E*[*n*]) compared to constant-shift invariant density functionals, but more of a balance for potential functionals. Note that in the case of *E*[*n*], the hypersensitivity stems from *E*_ext_ (the constant-shift dependent component). Since *E*_ext_ is exactly known, approximating it with ML accumulates unnecessary error.

We also construct a second dataset in which the hypersensitivity is intentionally avoided. This dataset is system-specific, containing a single molecule in which only the nuclear coordinates are varied. We show that within this dataset, all five quantities vary gradually with respect to the electron density, with ∣*ΔQ*/*Δn*∣ at or below the order of 1. Subsequently, all quantities are learned accurately regardless of the input. This highlights that the ‘learnability’ of a functional depends not only on the input and output variables, but on the steepness of the input-output mapping within the domain of the dataset. Our results suggest that the hypersensitivity we identify may not affect applications where system-specific datasets are used, such as molecular dynamics. System-specific datasets were used in ref. ^26^, which may partially explain why *E*[*n*] was learned more accurately than *E*[*v*_ext_]—we expect that the hypersensitivity of *E*[*n*] did not arise as the data did not explore the hypersensitive regions of the functional. In contrast, datasets containing a wide variety of systems—such as those used in ref. ^19^—are more likely to cover the hypersensitive regions of the mapping. As a consequence, the effect of hypersensitivity is evident in the results of ref. ^19^, explaining a significant portion of the error in their ML approximations to constant-shift dependent density functionals (*E*[*n*] and *E*_ext_[*n*]).

## Results

### Data generation

To obtain our training and testing data, we start by defining 1D molecular potentials of the form

1 $${v}_{{\rm{ext}}}(x)=-\frac{{Z}_{l}}{\sqrt{{\left(x+\frac{R}{2}\right)}^{2}+{\alpha }_{l}^{2}}}-\frac{{Z}_{r}}{\sqrt{{\left(x-\frac{R}{2}\right)}^{2}+{\alpha }_{r}^{2}}}.$$

(Atomic units are used throughout.) These potentials have the soft-core Coulomb form, which uses a softening parameter, *α* (since the 1D bare-Coulomb form has a non-integrable singularity)^40,41^. The reference level is fixed, as *v*_ext_(∣*x*∣ → *∞*) = 0 for all potentials. We construct two datasets, each comprising one hundred potentials. For the first dataset, we use a fixed internuclear separation of *R* = 5 and sample the remaining parameters uniformly in the following ranges: $3\le {Z}_{l}\le 3.5,\,1.25\le \frac{1}{{\alpha }_{l}}\le 1.75,\,1.5\le {Z}_{r}\le 2,\,1.25\le \frac{1}{{\alpha }_{r}}\le 1.75$. The external potentials are represented on a spatial grid of 75 points from *x* = − 7.5*a*_0_ to *x* = 7.5*a*_0_, ensuring that all relevant physical quantities are converged to within 10^−^^5^ Ha. For the second dataset, we fix both *Z*_*l*_ and *Z*_*r*_ at 2, and *α*_*l*_ and *α*_*r*_ at 1. We sample *R* uniformly in the range 2 ≤ *R* ≤ 7. For this dataset, we use a spatial grid of 70 points from *x* = −10*a*_0_ to *x* = 10*a*_0_ (due to the greater internuclear separations and our infinite boundary conditions, a grid with a larger spatial extent is required to achieve the same level of convergence).

For each external potential, we model systems of two electrons in a spin singlet (spin up and spin down), interacting via the soft-core Coulomb potential with *α* = 1, i.e. $U(x,x{\prime} )={\left({(x-x{\prime} )}^{2}+1\right)}^{-\frac{1}{2}}$. We numerically solve the many-body Schrödinger equation using the iDEA code^39^ to find the exact total energy, *E*, and the many-body wavefunction, from which we obtain the exact many-body density, *n*(*x*). We calculate the value of the HK universal functional for each system by subtracting the external potential energy from the total energy: *F* = *E* − *E*_ext_, where ${E}_{{\rm{ext}}}={\int }_{-\infty }^{\infty }{v}_{{\rm{ext}}}(x)n(x){\rm{d}}x$. To obtain the remaining quantities, we first solve the Schrödinger equation for the corresponding one- and three-electron systems. (For the three-electron systems, our electron spins are up, up, and down.) We then calculate the first ionisation energy, *I* = *E*_1_ − *E*_2_, the electron affinity, *A* = *E*_2_ − *E*_3_ and the fundamental gap, *E*_g_ = *I* − *A*.

### Dataset 1

We now illustrate the hypersensitivity effects described above using examples from our first dataset, where systems with similar electron densities differ substantially in their electronic properties.

In this dataset, variations in the nuclear charges and softening parameters of the external potentials lead to near-constant differences betweens potentials. An example of this is shown in Figure 2. The molecular potentials in panel (c) are defined by *Z*_*l*_ = 3.44, *α*_*l*_ = 0.65, *Z*_*r*_ = 1.64, *α*_*r*_ = 0.63 (solid black line) and *Z*_*l*_ = 3.10, *α*_*l*_ = 0.62, *Z*_*r*_ = 1.52, *α*_*r*_ = 0.63 (dashed orange line). Their difference, *Δv*_ext_(*x*), is also shown as a blue dotted line. *Δv*_ext_(*x*) is non-constant, peaking at *x* = ±*R*/2 and tending to 0 as ∣*x*∣ → *∞* (as necessitated by the fixed reference level). However, over the domain *x* = −3.9 to *x* = −1 (right), which contains over 97% of the electron density, it is approximately constant. As a result, the electron densities corresponding to the two potentials, *n*(*x*) and $\widetilde{n}(x)$, are very similar. This is visually apparent in panel (b), but we also attain a numerical measure using the *L*^2^ norm distance,

2 $$| | \widetilde{n}(x)-n(x)| {| }_{{L}^{2}}=\sqrt{{\int }_{-\infty }^{\infty }{\left[\widetilde{n}(x)-n(x)\right]}^{2}{\rm{d}}x},$$

and find that $| | \widetilde{n}(x)-n(x)| {| }_{{L}^{2}}=0.004$. Despite their proximity in density space, the difference in total energy is significant at $| \widetilde{E}-E| =0.54$. As the total energy responds significantly to the near-constant shift but the electron density does not, the total energy functional, *E*[*n*], is hypersensitive. In contrast, the value of the HK universal functional *F* differs by only 0.004 for the two systems, so *F*[*n*] is not hypersensitive, as anticipated. Since *E* = *F* + *E*_ext_, this analysis shows that the constant-shift dependent energy component, *E*_ext_, is responsible for the significant difference in total energy, not *F*. The near-constant shift also leads to large changes in *I* (0.27) and *A* (0.21), because they are constant shift dependent.

The rate at which a functional *Q*[*n*] changes with respect to the input can be approximated by the finite difference quotient

3 $$\left|\frac{\Delta Q}{\Delta n}\right|=\frac{| Q[\widetilde{n}]-Q[n]| }{| | \widetilde{n}(x)-n(x)| {| }_{{L}^{2}}}.$$

∣*ΔQ*/*Δn*∣ ≫ 1 indicates hypersensitivity, as ∣*Δn*∣ is small relative to ∣*ΔQ*∣. For the systems shown in Figure 2, ∣*ΔE*/*Δn*∣ = 122 and ∣*ΔF*/*Δn*∣ = 0.96.

Figure 3 shows a pair of systems from our dataset that exemplifies the hypersensitivity of our second class of functionals, i.e. any functional of the *N*-electron density that depends on the properties of the corresponding (*N* + 1)-electron system, such as *A*[*n*] and *E*_*g*_[*n*]. The potentials (c) are defined by *Z*_*l*_ = 3.49, *α*_*l*_ = 0.64, *Z*_*r*_ = 1.52, *α*_*r*_ = 0.77 (solid black line) and *Z*_*l*_ = 3.39, *α*_*l*_ = 0.63, *Z*_*r*_ = 1.52, *α*_*r*_ = 0.59 (dashed orange line). The two-electron densities (b) are concentrated in the left wells, where the difference between the potentials (blue dotted line) is close to 0. As a result, they essentially experience the same potential and differ by only 0.001 in *L*^2^. However, the difference between the potentials is significant in the right well, a region in which both systems have significant three-electron density (a). Consequently, *E*_*g*_ and *A* for the two systems differ by 0.39 and 0.32, respectively, with finite difference quotients of ∣*ΔE*_*g*_/*Δn*∣ = 318 and ∣*ΔA*/*Δn*∣ = 259. In contrast, ∣*ΔF*/*Δn*∣ = 0.83.

### Dataset 2

**Fig. 4:**
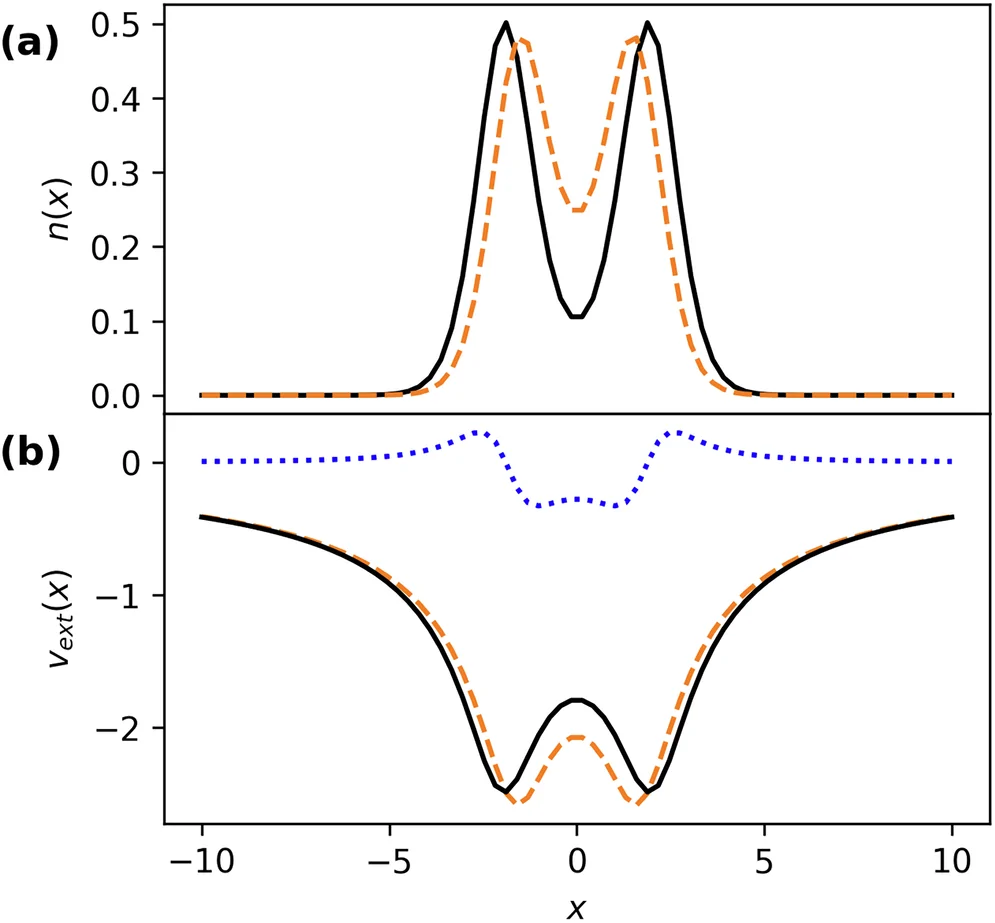
Two systems from our second dataset; the variation in the potentials in this dataset is such that our density functionals do *not* exhibit hypersensitivity. In this dataset, only the internuclear separation between the atomic wells, *R*, is varied—the external potential represented by a solid black line in (**b**) corresponds to *R* = 4, whereas the dashed orange line corresponds to *R* = 3.31. The blue dashed line shows the difference between the potentials, which varies significantly over the domain of the electron density (**a**). The electron density responds significantly to this change in potential, in contrast to the examples of hypersensitivity in Figs. 2 and 3.

Figure 4 shows an example pair of systems from our second dataset, in which only the internuclear separation, *R*, is varied. The systems represented by solid black lines and dashed orange lines correspond to *R* = 4 and *R* = 3.31, respectively. In contrast to Figs. 2 and 3, the difference between the two external potentials (blue dotted line) has significant spatial variation over the domain of the electron density. As a consequence, the two systems have significantly different electron densities (*L*^2^ = 0.36), and the hypersensitivity issues do not arise: for example, ∣*ΔE*/*Δn*∣ = 0.56 and ∣*ΔA*/*Δn*∣ = 0.08.

### Estimating the local Lipschitz constant

We now extend our analysis to each dataset as a whole, considering all pairs of systems *i* and *j*, *i* ≠ *j*. For each pair, we compute the finite difference quotient ∣*ΔQ*/*Δn*∣ for all quantities. We then take the maximum finite difference quotient for each quantity as an empirical estimate of the local Lipschitz constant, $\widehat{L}$^42^.

4 $$\widehat{L}=ma{x}_{i\ne j}\frac{| Q[{n}^{i}]-Q[{n}^{j}]| }{| | {n}^{i}(x)-{n}^{j}(x)| {| }_{{L}^{2}}}$$

$\widehat{L}$ is a lower bound on the maximum rate of change within the domain of the dataset. It is commonly used as a measure of learning complexity^43^, e.g. to attain bounds for the worst-case prediction error^42^. We use it to characterise the degree of hypersensitivity of each functional in our datasets.

**Fig. 5:**
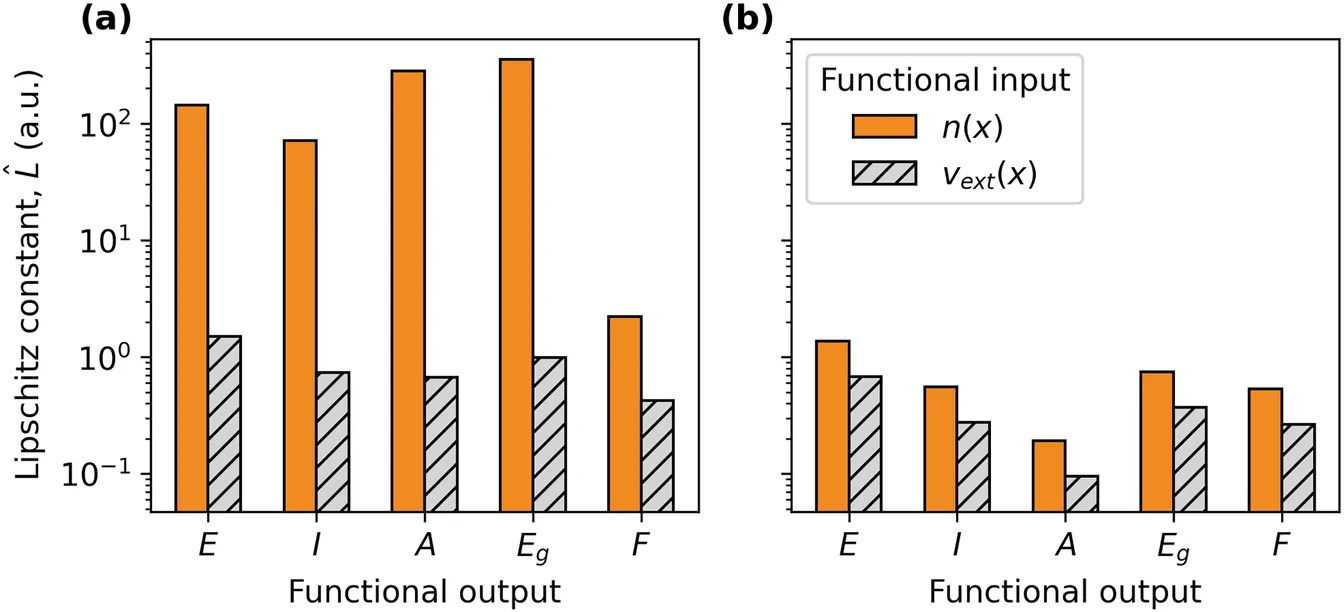
The local Lipschitz constant, $\widehat{L}$, is plotted for all density functionals (orange bars) and potential functionals (grey hatched bars), enabling a comparison of how rapidly each varies with respect to its input. As $\widehat{L}$ represents (a lower bound on) the maximum rate of change within a restricted domain, it has different values in our two datasets. In our first dataset (**a**), the hypersensitivity effects we have identified manifest as values of $\widehat{L}$ on the order of 10^2^ for *E*[*n*], *I*[*n*], *A*[*n*] and *E*_*g*_[*n*]. For *F*[*n*], which does not suffer from these effects, $\widehat{L}$ is two orders of magnitude smaller. In our second dataset (**b**), none of the density functionals exhibit hypersensitivity, as expected. Moreover, in both datasets, $\widehat{L}$ is small for all potential functionals, indicating the absence of hypersensitivity.

In Figure 5 we plot $\widehat{L}$ for all density functionals (solid orange bars) in our datasets. In the first dataset (a), $\widehat{L}\,\gg \,1$ for *E*[*n*], *I*[*n*], *A*[*n*] and *E*_*g*_[*n*], providing numerical confirmation of the hypersensitivity described above. (b) shows that, as expected, the same density functionals are *not* hypersensitive in our second dataset, as $\widehat{L}$ is on the order of 10^−1^−10^0^. We also plot $\widehat{L}$ for each quantity as a functional of the external potential (grey hatched bars), showing that the hypersensitivity does not arise for potential functionals in either dataset. This is because the small changes in electron density in general correspond to significant changes in the external potential (for example, see Figs. 2 and 3), so ∣*ΔQ*/*Δv*_ext_∣ is consistently small.

### Machine learning

The ML model we use is KRR; see section ‘Kernel Ridge Regression’ for details. To assess the model’s accuracy, we use the mean absolute error (MAE)

5 $${\rm{MAE}}=\frac{1}{S}\mathop{\sum }\limits_{i=1}^{S}| {Q}^{{\rm{ML}}}[{n}^{i}]-Q[{n}^{i}]| ,$$

where *n*^*i*^ are the inputs in the test set, of which there are *S* = 10 (note that since we use nested k-fold cross-validation, all 100 samples are used for testing at some point).

**Fig. 6:**
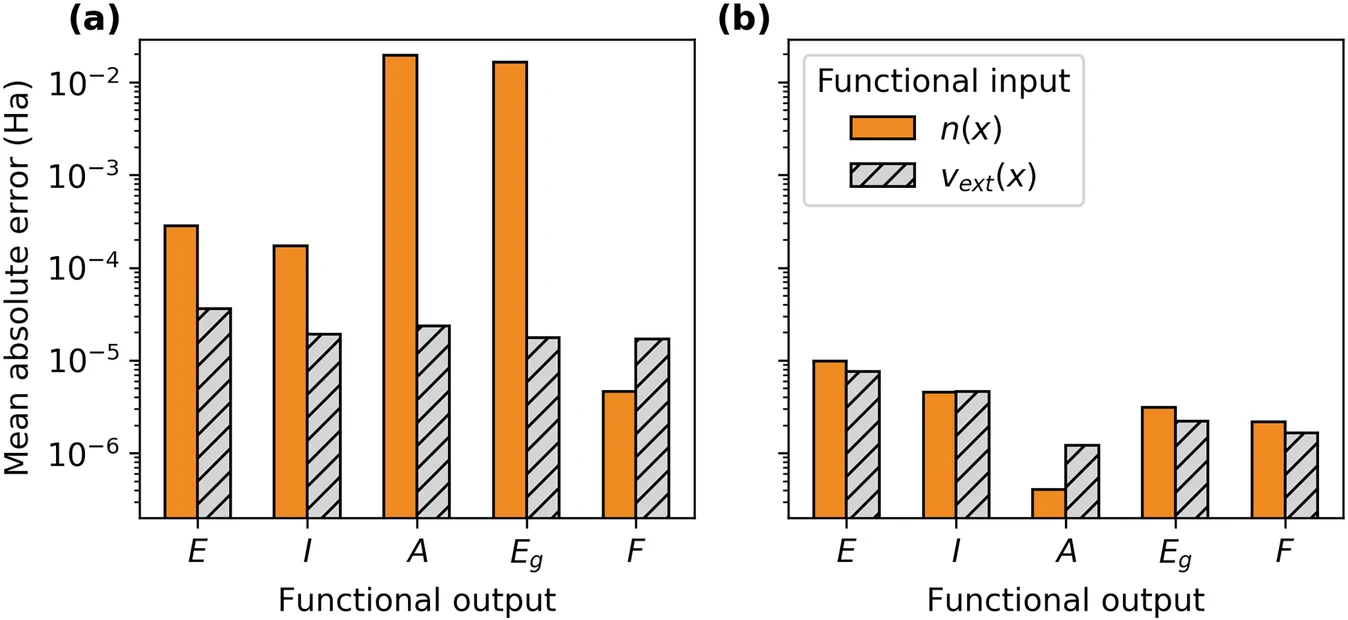
The MAE in our ML approximations to density functionals (orange bars) and potential functionals (grey hatched bars). Comparing to Fig. 5, it is apparent that the ML accuracy is closely related to the Lipschitz constant, i.e. rapidly-varying functionals are approximated less accurately. Our hypotheses account for three key aspects of these results: for the first dataset (**a**), the ML approximations to the hypersensitive functionals *E*[*n*], *I*[*n*], *A*[*n*] and *E*_*g*_[*n*] are less accurate than the *F*[*n*] approximation, as *F*[*n*] is neither constant-shift dependent nor does it depend on the properties of the (*N* + 1)-electron system. In the second dataset (**b**), none of the functionals exhibit hypersensitivity, and hence the ML approximations are similarly accurate. Discrepancies in ML accuracy do not occur when the external potential is used as the input, because the potential functionals are not hypersensitive in either dataset.

Figure 6 shows the MAE in the KRR predictions of each physical quantity when using the electron density (solid orange bars) and the external potential (hatched grey bars) as input. First, we will discuss the results for Dataset 1 (the dataset in which the density functionals exhibit hypersensitivity), which are shown in panel (a). Amongst the density functionals, the error is one to three orders of magnitude greater for the functionals we identified as hypersensitive (*E*[*n*], *I*[*n*], *A*[*n*] and *E*_*g*_[*n*]). For example, although the error in *E*[*n*] was very low in absolute terms (0.28 mHa or 0.18 kcal/mol) (due to the quality of data provided by our 1D systems), it was 61× greater than the error in *F*[*n*] (4.65 × 10^−3^ mHa or 2.9 × 10^−3^ kcal/mol). This means that the vast majority of the error in *E*[*n*] would be avoided by calculating *E*_ext_[*n*] via the closed-form expression rather than attempting to machine-learn it. The errors in *A*[*n*] and *E*_*g*_[*n*] were the greatest by a significant margin at 19.4 and 16.4 mHa, respectively—around 4000× greater than the error in *F*[*n*]. This is likely because these quantities, due to their dependence on the (*N* + 1)-electron energy, are the most sensitive in the dataset (note that they have the greatest values of $\widehat{L}$ in Fig. 5).

*E*, *I*, *A* and *E*_*g*_ are all predicted with significantly greater accuracy from the external potential, because the hypersensitivity issue is avoided. Specifically, we observe about a 10-fold decrease in error for *E* and *I*, and a 1000-fold decrease for *A* and *E*_*g*_, when using the external potential as input rather than the electron density. This confirms that the pathologies of the density → output mapping are the source of error, not the output quantities per se.

These results are in striking agreement with ref. ^19^, where ML was employed to predict the total energy, external potential energy, exchange, correlation and kinetic energy of electrons in random 2D potentials. When using the electron density as input to their ML models, the authors observed significantly greater error for the total energy and external potential energy relative to the exchange, correlation, and kinetic energy; the MAE was typically an order of magnitude greater. We posit that this is explained by hypersensitivity, as *E* and *E*_ext_ are constant shift-dependent and the other quantities are not. However, the authors attributed it to the fact that their dataset contained a larger range of values for *E* and *E*_ext_ compared to the other quantities. As this explanation relies solely on the target property, it implies that the discrepancy in predictive accuracy should not depend on the input to the ML model. However, when the authors used the external potential as the input, the error was more balanced between the different properties. Likewise, we find that when using the external potential as the input, our KRR model is equally capable of predicting constant-shift dependent quantities and constant-shift invariant quantities; see Fig. 6a. Our hypersensitivity hypothesis explains why the discrepancy occurs for density functionals and not potential functionals. In addition, as the authors of ref. ^19^ used NNs, while we used KRR, this implies the difficulties of learning these density functionals are independent of the ML method. Figure 6b shows the results for Dataset 2. This dataset is system-specific (only the internuclear separation is varied), and all observables vary gradually with respect to the electron density. As the hypersensitivity of *E*[*n*], *I*[*n*], *A*[*n*] and *E*_*g*_[*n*] is intentionally avoided in this dataset, the resulting ML approximations are far more accurate. This highlights that the difficulty of learning a functional depends critically on its local behaviour within the domain of the data. Ref ^44^ similarly found that ML was more challenging in the region of a quantum phase transition, where the functional is non-analytic. Our results suggest that the hypersensitivity effects we identified may not arise for applications where system-specific datasets are used, such as molecular dynamics simulations. This also explains why in ref. ^26^, in which only system-specific datasets were used, the external potential was not a better input than the electron density for predicting the total energy; since each dataset only consisted of a single molecule, we hypothesise that the hypersensitivity of *E*[*n*] did not arise. However, while system-specific functionals are useful for certain applications, the central aim of ML-DFT is to train a model that can reliably generalise across diverse physical systems^17^. This requires a broad and varied dataset, so the hypersensitivity is much more likely to arise. As ref. ^19^ used a very broad dataset, it is not surprising that these effects were encountered. This is why an alternative solution, such as using the external potential as input, is required.

Although our findings reveal a distinct advantage to using the potential rather than the density in specific contexts, we note that there may be scenarios in which the potential is the inferior input. Like density functionals, potential functionals may also exhibit hypersensitivity, non-analyticity, and other pathologies that make ML challenging. Moreover, for some systems, the potential to observable mapping is one-to-many, such as an open-shell atom or molecule with orbital degeneracy, for which the external potential maps to several distinct, degenerate densities. Hence, when selecting which input to use, the uniqueness and numerical behaviour of the input-output mapping must be considered. In this paper, we find that for a variety of important observables with well-defined mappings in both DFT and PFT, hypersensitivity affecting the density functionals is absent in the corresponding potential functionals, demonstrating a clear advantage to the potential-based approach in the contexts we outline.

## Discussion

Although in principle, ML models such as KRR and NNs can learn the mapping from the electron density to any electronic property, some physical quantities can be highly sensitive to small variations in the electron density, which makes accurate ML far more challenging. In this paper, we identified two sources of this ‘hypersensitivity’ within DFT, each affecting an entire class of observables: (1) quantities that vary when a constant shift is applied to the external potential and (2) functionals of the *N*-electron density that depend on the properties of the (*N* + 1)-electron system.

The hypersensitivity is caused by changes to the external potential of a system, *Δv*_ext_(***r***), that have only a negligible impact on its electron density but a disproportionately large impact on the target quantity. This occurs in the first class of functionals when *Δv*_ext_(***r***) is near-constant over the region where the electron density is concentrated (which is possible even when the reference level of the external potential is fixed). Hypersensitivity arises in the second class of quantities when *Δv*_ext_(***r***) has small spatial variation over the domain of the *N*-electron density but is significant in the domain of the (*N* + 1)-electron density. In both cases, *Δv*_ext_(***r***) itself is not small, so the observables are not hypersensitive to these changes in the potential.

Using exactly solvable one-dimensional model systems that form the basis of our ML datasets, we provide examples of these effects, where pairs of systems with near-identical electron densities differ substantially in their total energy (Class 1), first ionisation energy (Class 1), electron affinity (Classes 1 and 2), or fundamental gap (Class 2). We find that with a fixed dataset size and training procedure, KRR models exhibit significantly greater error when applied to these functionals compared to the HK universal functional, which does not suffer from the sources of hypersensitivity we identified. Both in this research and previous work which employed NNs^19^, the external potential energy, *E*_ext_[*n*], has proven to be the most challenging component of the total energy to learn as a density functional. Our analysis explains this surprising result; although *E*_ext_[*n*] has a simple, closed form, it is hypersensitive when expressed as a pure density functional and hence highly complex from an ML standpoint. Since *E*_ext_ can be calculated via a closed-form expression, our findings highlight that it is preferable to machine-learn *F*[*n*] and then add the external potential energy contribution, rather than attempting to learn the mapping from the density to the total energy directly.

For all observables in Classes 1 and 2, we found that the hypersensitivity can be avoided by using the external potential as input to the ML model. With this approach, we attained considerably more accurate predictions of the total energy, first ionisation energy, electron affinity and fundamental gap. This also explains why, in ref. ^19^, the error was more balanced between constant-shift dependent and constant-shift invariant quantities when using the external potential as input than when using the electron density.

We also provide a second example dataset which restricts the variations between systems so that density functionals in Classes 1 and 2 do *not* exhibit hypersensitivity, and are consequently learned with far greater accuracy. Note that it is not that these functionals are not hypersensitive on the whole, but rather the dataset does not explore the hypersensitive regions of the mapping. This highlights the dataset-dependent nature of the problem and raises the question: in which contexts is it likely to arise? As our second dataset is system-specific (containing only a single 1D molecule), we hypothesise that the hypersensitivity is unlikely to arise in system-specific datasets; this would also explain why it does not seem to have arisen in ref. ^26^. On the other hand, in a typical ML-DFT dataset that contains a broad range of physical systems, there are more likely to be near-constant differences between potentials (for example), and hence hypersensitivity.

Although our numerical examples are one-dimensional, our arguments readily extend to two- and three-dimensional systems. Moreover, while we have focused on two specific sources of hypersensitivity, there are likely to be other cases that follow the same pattern, i.e. the observable is sensitive to some change in the potential but the density is not. As such, our solution of using the external potential as input to the ML model could also circumvent other possible sources of hypersensitivity in density functionals that we have not identified in this paper.

Future research could apply our methodology to identify hypersensitivity in realistic ML-DFT datasets and to determine whether our proposed potential-based approach could be beneficial. As such, our findings offer the prospect of reliable ML models within DFT and PFT for a variety of important physical quantities that may, in turn, lead to more robust computational modelling within materials science.

## Methods

### Kernel ridge regression

KRR is a non-linear regression model with regularisation^22^. To approximate a functional *Q*[*n*], KRR uses a kernel function *k*[*n*^*i*^, *n*^*j*^] that measures the similarity of two inputs *n*^*i*^ and *n*^*j*^. We use the Gaussian kernel, $k[{n}^{i},{n}^{j}]=\exp (-| | {n}^{i}-{n}^{j}| {| }_{{L}^{2}}^{2}/(2{\sigma }^{2}))$, a standard choice in ML-DFT^20,22,26,45^. For a given input, *n*, the predicted value, *Q*^ML^[*n*], is calculated as a weighted sum of the similarity scores of *n* with each *n*^*j*^ in the training set^45^, of which there are *M*, i.e.

6 $${Q}^{{\rm{ML}}}[n]=\mathop{\sum }\limits_{j=1}^{M}{\alpha }^{j}k[n,{n}^{j}].$$

The weights ${\boldsymbol{\alpha }}={({\alpha }^{1},\ldots ,{\alpha }^{M})}^{T}$ are found by minimising the cost function

7 $$C({\boldsymbol{\alpha }})=\mathop{\sum }\limits_{j=1}^{M}{({Q}^{{\rm{ML}}}[{n}^{j}]-Q[{n}^{j}])}^{2}+\lambda | | Q| {| }_{{{\mathcal{H}}}_{c}}^{2},$$

where $| | Q| {| }_{{{\mathcal{H}}}_{c}}^{2}={\boldsymbol{\alpha }}\cdot {\mathbb{K}}{\boldsymbol{\alpha }}$ is the reproducing kernel Hilbert space (RKHS) norm^46^ and ${\mathbb{K}}$ is a matrix with elements *K*_*ij*_ = *k*[*n*^*i*^, *n*^*j*^]. The greater the value of the regularisation parameter *λ*, the more emphasis is placed on minimising the RKHS norm, which can prevent the model from overfitting the training data.

The cost function is minimised by solving ${\boldsymbol{\alpha }}={({\mathbb{K}}+\lambda {\mathbb{I}})}^{-1}{\boldsymbol{Q}}$, where ${\mathbb{I}}$ is the identity matrix and ${\boldsymbol{Q}}={({Q}^{1},\ldots ,{Q}^{M})}^{T}$ are the outputs (or ‘labels’) in the training set^45^. Note that both *λ* and *σ* are hyperparameters, meaning their values are chosen explicitly before training. The small number of hyperparameters in KRR makes optimising (or ‘tuning’) them simpler than in more complex models such as NNs. Training is also easier for KRR due to the closed-form solution, which finds the global minimum of the loss function. In contrast, there is no such guarantee for NNs, which are typically trained via stochastic optimisation methods. As such, we employ KRR in this paper as the technique is less dependent upon user input than NNs, offering a fairer demonstration of our hypotheses. However, we note that our findings are not specific to KRR, as similar results have been reported in previous work using NNs; see ref. ^19^ (discussed in section ‘Results’).

To tune and test our KRR models, we use nested k-fold cross-validation in combination with grid searches^47^. The nested validation comprises 10 outer folds and 9 inner folds with an 80-10-10 train-validation-test split, providing robust measures of model performance both at the validation and testing stages. Previous studies have shown that KRR can learn highly accurate DFAs from a small number of training samples^21^, and may outperform NNs in the small-data regime^47^.

## Data Availability

The datasets generated and analysed during the current study are openly available in the research data repository of the University of York, https://doi.org/10.15124/be174a35-5ae0-4758-85f1-d21d7cffdeba.

## Code availability

The underlying code for this study is available in the iDEA-org GitHub repository and can be accessed via this link https://github.com/iDEA-org/iDEA.
